# The Re-Emergence and Emergence of Vector-Borne Rickettsioses in Taiwan

**DOI:** 10.3390/tropicalmed3010001

**Published:** 2017-12-21

**Authors:** Nicholas T. Minahan, Chien-Chung Chao, Kun-Hsien Tsai

**Affiliations:** 1 Institute of Environmental Health, College of Public Health, National Taiwan University, No. 17, Xu-Zhou Road, Taipei 100, Taiwan; f05841021@ntu.edu.tw; 2 Viral and Rickettsial Diseases Department, Infectious Diseases Directorate, Naval Medical Research Center, Silver Spring, MD 20910, USA; chien-chung.c.chao.civ@mail.mil; 3 Department of Public Health, College of Public Health, National Taiwan University, No. 17, Xu-Zhou Road, Taipei 100, Taiwan

**Keywords:** vector-borne rickettsioses (VBR), scrub typhus, murine typhus, spotted fever group rickettsiae, *Rickettsia felis*, Anaplasmataceae, re-emerging, emerging, Taiwan

## Abstract

Rickettsial diseases, particularly vector-borne rickettsioses (VBR), have a long history in Taiwan, with studies on scrub typhus and murine typhus dating back over a century. The climatic and geographic diversity of Taiwan’s main island and its offshore islands provide many ecological niches for the diversification and maintenance of rickettsiae alike. In recent decades, scrub typhus has re-emerged as the most prevalent type of rickettsiosis in Taiwan, particularly in eastern Taiwan and its offshore islands. While murine typhus has also re-emerged on Taiwan’s western coast, it remains neglected. Perhaps more alarming than the re-emergence of these rickettsioses is the emergence of newly described VBR. The first case of human infection with *Rickettsia felis* was confirmed in 2005, and undetermined spotted fever group rickettsioses have recently been detected. Taiwan is at a unique advantage in terms of detecting and characterizing VBR, as it has universal health coverage and a national communicable disease surveillance system; however, these systems have not been fully utilized for this purpose. Here, we review the existing knowledge on the eco-epidemiology of VBR in Taiwan and recommend future courses of action.

## 1. Introduction

Taiwan is located in East Asia off the southeastern coast of China, intersected by the Tropic of Cancer, which divides the coastal lowlands into subtropical and tropical climates. Taiwan’s terrain is dominated by a central mountain range with a temperate climate, which is sparsely inhabited. Most of Taiwan’s population is concentrated in metropolitan areas in the north (Taipei basin) and southward along the western coast. Yet, it is Taiwan’s offshore islands and eastern region that have historically been, and remain, the hyperendemic foci of vector-borne rickettsioses (VBR).

VBR are caused by rickettsiae, obligate intracellular Gram-negative bacteria belonging to the Rickettsiaceae and Anaplasmataceae families within the Rickettsiales order, including the genera *Rickettsia*, *Orientia*, *Ehrlichia*, and *Anaplasma* [1]. VBR have complex life cycles, involving vertebrate reservoir hosts and hematophagous arthropod vectors (ticks, fleas, mites, and lice) that also serve as the main reservoir host for some VBR, as is the case for chigger (*Leptotrombidium* spp.) mites in the maintenance of *Orientia tsutsugamushi* [2], the etiologic agent of scrub typhus, and cat fleas (*Ctenocephalides felis*) in the maintenance of *Rickettsia felis* [3]. Humans are incidental (dead-end) hosts for most VBR, which cause acute undifferentiated febrile illnesses that are potentially life-threatening if untreated [4]. While scrub typhus is the most common type of rickettsiosis in Taiwan, murine typhus, a type of flea-borne rickettsiosis caused by *Rickettsia typhi*, is another important endemic form of VBR. These two types of rickettsioses belong to the scrub typhus group (STG) and typhus group (TG), respectively, and constitute re-emerging VBR in Taiwan. In contrast, most emerging VBR are tick-borne and belong to the spotted fever group (SFG), caused by various *Rickettsia* species, with the exception of flea-borne spotted fever, caused by *R. felis*, and mite-borne rickettsialpox, caused by *Rickettsia akari* (absent in Taiwan), which both belong to the transitional group (TRG) [5]. Epidemic typhus is a louse-borne rickettsiosis caused by *Rickettsia prowazekii* that also belongs to the TG and is the only VBR known to latently infect humans [4]; however, it has not occurred in Taiwan since World War II (WWII). However, *R. prowazekii* is classified as a category B bioterrorism agent by the U.S. Centers for Disease Control [6] and is a category II notifiable disease in Taiwan [7].

### Historical Review

**Figure 1:**
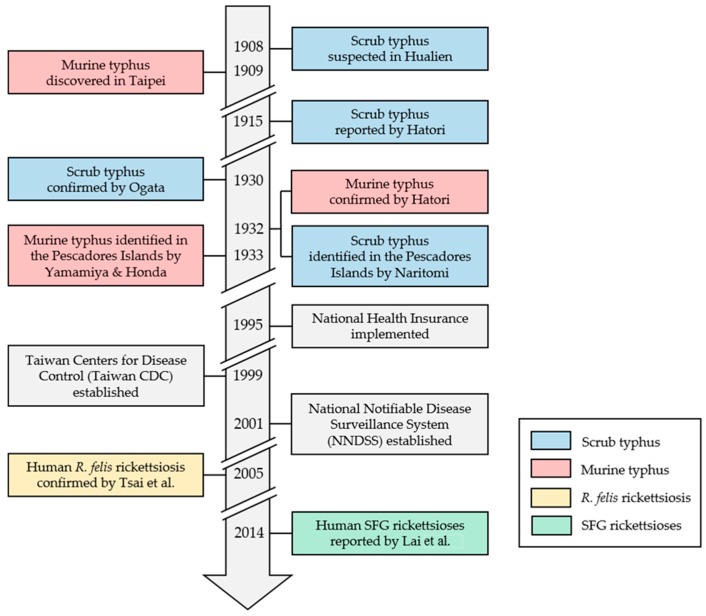
Historical review of human vector-borne rickettsioses (VBR) and recent public health milestones in Taiwan [7,8,9,10,11,12,13,14,15,16,17]. SFG: spotted fever group; *R. felis*: *Rickettsia felis.*

Scrub typhus (tsutsugamushi disease) was first suspected among Japanese police officers in Hualien in 1908 [8,9,10], and murine typhus was discovered in Taipei in 1909 [10]. Jūrō Hatori was the first to draw a parallel between various reports of an unknown fever to tsutsugamushi disease in 1915 [11], recognizing the role of red (*Leptotrombidium* spp.) larval mites in disease transmission. Japanese physicians and scientists continued to characterize scrub typhus in Taiwan through WWII, especially in the Pescadores Islands, whereas few studies described murine typhus (Figure 1).

After the Korean War, the U.S. Naval Medical Research Unit No. 2 (NAMRU-2) was re-established in Taipei [18], which served as the regional headquarters through the Vietnam War. Recognized as a hyperendemic focus of scrub typhus, the Pescadores Islands remained the site of most NAMRU studies, especially among military personnel [19,20,21,22]. By 1967, Lien et al. [23] established *Leptotrombidium deliense* as the vector for scrub typhus in the Pescadores Islands. Then, Gale et al. [24] isolated *O. tsutsugamushi* from free-living *L. deliense* and wild rats (*Rattus* species) captured near an outbreak in eastern Taiwan among military personnel in 1970. Notably, Olson et al. published a series of papers describing the eco-epidemiology of scrub typhus in the Pescadores Islands, correlating monthly mean temperature and monthly incidence of scrub typhus from 1973 to 1976 [25], generating a model to forecast scrub typhus epidemics [26], and elucidating socioeconomic factors (increased urbanization and increased school enrollment) associated with the decreased incidence of scrub typhus [27]. Olson et al. also demonstrated the prophylactic efficacy of doxycycline (200 mg weekly) for scrub typhus through a randomized double-blind study among 1125 military subjects in the Pescadores Islands [28].

## 2. Re-Emerging Vector-Borne Rickettsioses

### 2.1. Scrub Typhus

#### 2.1.1. Epidemiology of Scrub Typhus

**Figure 2:**
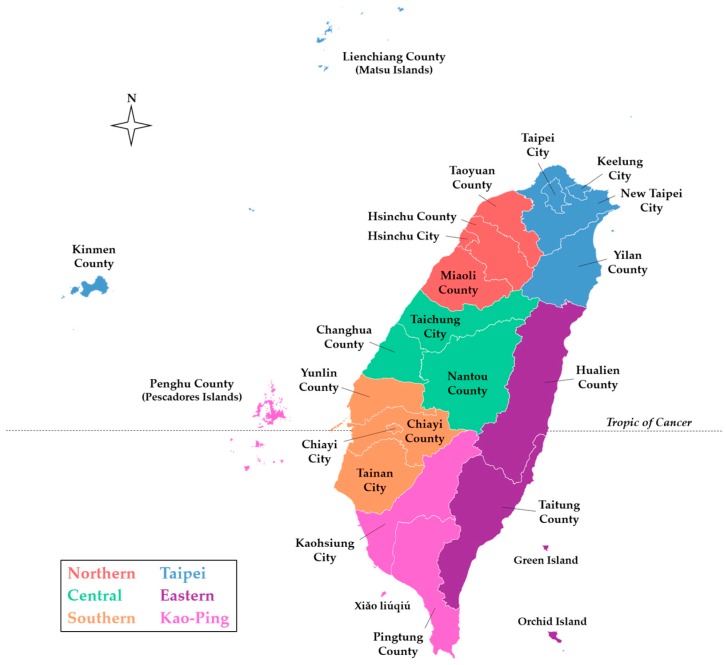
County-level map of Taiwan indicating the six regions of the Taiwan CDC NNDSS [7]. The basemap was retrieved from the Taiwan Ministry of the Interior [33].

**Figure 3:**
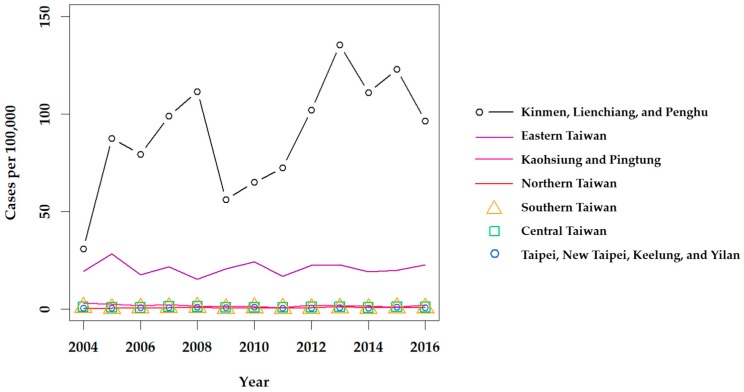
Annual prevalence of scrub typhus throughout Taiwan from 2004 to 2016. Confirmed indigenous cases were retrieved from the Taiwan CDC NIDSS [32]. 2010 Population and Housing Census data, retrieved from the Taiwan Statistics Bureau [34], were used to calculate the prevalence.

Although scrub typhus was designated as a notifiable disease in 1955 [29], it is difficult to estimate how many cases were in the general population prior to 1990. Lee et al. reported that the annual number of confirmed cases of scrub typhus increased from 39 in 1990 to 302 in 1999 [30], and revealed a significant increase among confirmed cases in eastern Taiwan from 2001 to 2004 [31]. Scrub typhus has remained the most prevalent type of rickettsiosis in Taiwan in recent years, with a mean of 435 cases confirmed annually from 2004 to 2016 [32]. Clinically suspected cases of scrub typhus, as well as murine typhus, are mandatorily reported to each Taiwan CDC regional center, and biological specimens (i.e., whole blood or serum) are sent to the Taiwan CDC Vector-borne Viral and Rickettsial Diseases laboratory in Taipei and screened by immunofluorescence assay (IFA) for diagnosis, confirmed by an immunoglobulin M (IgM) titer of 1:80, IgG titer of 1:320, or a 4-fold increase of IgG between acute and convalescent phase paired sera. Definitive diagnosis is also made if *O. tsutsugamushi* is isolated or detected by polymerase chain reaction (PCR). Then, confirmed cases are reported through the regional center back to the reporting hospital (Figure 2). From 2004 to 2016, the annual prevalence of confirmed indigenous cases of scrub typhus was the highest in the eastern region of the Taiwan CDC NNDSS; however, stratification of the Taipei and Kaohsiung-Pingtung (Kao-Ping) regions reveals a much higher prevalence in the Kinmen, Lienchiang, and Penghu Counties (Figure 3).

Due to the geographic and climatic diversity of Taiwan, it is difficult to interpret the effects of seasonal and meteorological factors on the incidence of scrub typhus. Thus, Tsai and Yeh [35] divided Taiwan and its offshore islands (excluding Xiǎo liúqiú, Green, and Orchid Islands) into 10 local climate regions, applied geographically weighted regression, and revealed that surface temperature correlates positively with the incidence of scrub typhus in central western and southwestern Taiwan, Kinmen, Matsu, and the Pescadores Islands from 2002 to 2011. Further, a positive correlation between scrub typhus incidence and precipitation was observed in central western and southwestern Taiwan and Kinmen [35]. They also found that island-wide incidence of scrub typhus was significantly higher in the warm season (May to October) as compared to the cold season (November to April), with peaks occurring in June and July [35]. Chen et al. [36] used a Poisson generalized additive mixed model to determine the relative risk (RR) of scrub typhus after varying rainfall events (regular <130 mm, heavy 131–200 mm, torrential 201–350 mm, and extreme torrential >350 mm) from 1994 to 2008, revealing a significant positive trend (*p* = 0.001) with a RR of 1.782 (95% confidence interval 1.089, 2.915) after torrential rainfall at lag day 21.

A recent systematic review attempting to estimate the global incidence and burden of scrub typhus [37] inadequately described the epidemiology of scrub typhus in Taiwan. The authors extrapolated the incidence of scrub typhus in Taiwan from a study conducted exclusively in eastern Taiwan from 2000 to 2004 [31], which, as demonstrated in Figure 3, does not provide an island-wide representation. Kuo et al. [38] analyzed cases of scrub typhus on the main island (excluding offshore islands) from 2003 to 2008 and reported a mean incidence rate (IR) of 1.15 cases per year per 100,000 with significantly higher incidence among males as compared to females (*p* <0.001) and the highest age-specific incidence among those aged 60–69 years. This study also revealed that the proportion of farmers in the population, particularly dry-field farmers, is a significant positive predictor of scrub typhus standardized IR [38]. Further, Tsai and Yeh [35] revealed that the percentage of farm workers was a significant positive predictor of scrub typhus incidence in low-land townships in Hualien and Taitung Counties, the central mountainous and southern township areas, and Heping Township of Taichung County.

#### 2.1.2. Clinical Features of Scrub Typhus

In Taiwan, scrub typhus typically presents with fever (90–100%) [31,39] and, less consistently, chills (31–81%) [30,40], cough (20–72%) [40,41], lymphadenopathy (11–75%) [31,42], headache (21–63%) [30,39], and rash (8–56%) [40,41]. Eschar, a black scab that may appear at the site of the chigger bite after a painless papule ulcerates, is identified less frequently (23–65%) [31,43] on the main island of Taiwan as compared to other endemic countries (e.g., 92% in Korea [44] and 97% in Japan [45]); this is usually attributed to the extent of physical examination. Interestingly, however, eschars are observed in most cases of scrub typhus on the offshore islands. In fact, Yamamiya first made this distinction in 1933 [46], noting that eschars were always present on patients in the Pescadores Islands, whereas ‘atypical’ cases without eschars appeared on the main island. Even earlier, in 1916, Hatori only found eschars on 40 of 82 patients in Pingtung and Hualien [47]. Su et al. [40] reported eschars in 93% of 261 cases from 2005 to 2008 in Kinmen; however, studies of scrub typhus on the offshore islands in recent years are lacking, and an explanation for the regional variation in eschar formation remains elusive. Often overlooked, relative bradycardia, which has been defined in Taiwan as a heart rate of <110 beats per minute with a fever ≥38.9 °C without medication that alters heart rate, has recently been identified as a useful clinical feature for the differential diagnosis of scrub typhus in Korea [48]. Although evidence is limited, Lee et al. [30] and Su et al. [40] observed relative bradycardia in 11 of 15 patients (73%) and 95 of 137 patients (69%), respectively, consistent with the recent finding in Korea [48].

Once suspected, scrub typhus is readily treated with doxycycline [49]. However, in elderly, those with co-morbidity, or those with delayed time to treatment, severe complications occasionally occur. Pneumonitis was seen in 12 of 33 cases (36%) and 9 of 16 cases (56%) described by Tsay et al. [39] and Lee et al. [30], respectively, who also reported markedly high rates of acute respiratory distress syndrome (ARDS) (15–19%) and acute renal failure (ARF) (6–9%), among other serious complications, including disseminated intravascular coagulation (DIC), septic shock, meningitis, myocarditis, and acute pancreatitis in 5, 4, 2, 1, and 1 cases, respectively, between studies. Wang et al. [43] also reported ARDS in 8 of 72 cases (11%) seen in southern Taiwan from 1998 to 2006. Larger, more representative studies such as those of Su et al. [40] and Chang et al. [50], who described 187 cases in southern Taiwan from 2002 to 2011, reported lower rates of ARF (2–3%), ARDS (1–3%), DIC (0–3%), septic shock (1–2%), and acute pancreatitis (0–2%).

Together, these studies suggest a mortality rate of approximately 1% for patients with scrub typhus in Taiwan (5 deaths among 569 cases), representing 22% of those who developed ARDS [30,39,40,43,50]. It is important to note that 108 of 261 patients in Su et al. [40] were aged 20 to 29 and that 2 deaths occurred in patients with chronic obstructive pulmonary disorder complicated by acute hepatic failure [43]. Favorable clinical outcome in patients with scrub typhus relies on physician vigilance, ensuring rapid diagnosis and effective treatment.

#### 2.1.3. Ecology of Scrub Typhus

In recent years, several studies have investigated the ecology of scrub typhus (*O. tsutsugamushi*), among other VBR, examining small mammals (rodents and shrews) and their ectoparasites (chiggers, in the case of scrub typhus) throughout Taiwan (Table 1). Importantly, Kuo et al. [51] analyzed *O. tsutsugamushi* in small mammals and ectoparasitic chiggers in a far-reaching field survey throughout Taiwan (Yilan, Hualien, Taitung, Taoyuan, Taichung, and Kaohsiung-Pingtung), including Matsu, Kinmen, and the Pescadores and Orchid Islands, between 2006 and 2010. This study revealed that *L. deliense* is the most common chigger species at most study sites year-round (65% of chiggers identified overall), except in Hualien (51% *Leptotrombidium imphalum*), also replaced by *Leptotrombidium scutellare* in Kinmen during the cold season, whereas Matsu harbored diverse chigger species that varied by season, including *L. deliense* (76%) and *Leptotrombidium kawamurai* (24%) in the warm season and *Leptotrombidium pallidum* (80%), *L. scutellare* (12%), and *Leptotrombidium yui* (8%) in the cold season [51]. The lesser rice-field rat (*Rattus losea*) was the most abundant and widespread rodent (49% of total captures, present at all sites), while the Asian house shrew (*Suncus murinus*) and the rice-field rat (*Mus caroli*) accounted for 21% and 11% of total captures (*n* = 1284), respectively [51]. Of note, the Asian house rat (*Rattus tanezumi*) and the Polynesian rat (*Rattus exulans*) were captured exclusively in Orchid Island and Hualien, respectively [51]. *R. tanezumi* was the dominant species in Orchid Island, which demonstrated the highest *O. tsutsugamushi* seroprevalence among any rodent or shrew species in this study (92%), as well as the highest prevalence of chigger infestation (100%), chigger loads (mean of 394 per host), and chigger *O. tsutsugamushi* PCR positivity rate (96%) [51]. Lastly, a detailed analysis of *R. losea* revealed that *R. losea* captured in counties with a higher incidence of scrub typhus had significantly higher *O. tsutsugamushi* seroprevalence and prevalence and loads of chigger infestation, but not chigger *O. tsutsugamushi* PCR positivity rate [51].

In a similar study, Lin et al. [60] captured small mammals and collected ectoparasitic chiggers across Taiwan (Tainan, Hualien, and Taitung) and its offshore islands (Matsu, Kinmen, and the Pescadores Islands) between 2006 and 2010. Similar to Kuo et al. [51], *S. murinus* represented 35% of total captures; however, *R. losea* represented only 14% of total captures (*n* = 1061), nevertheless remaining the most common rodent species (28% of rodents) [60]. Also consistent with Kuo et al. [51], *R. exulans* was captured exclusively in Hualien, although *R. losea* and *Bandicota indica* were more common in Hualien in this study [60]. Other predominant rodent species identified in this study included *Rattus flavipectus* (19% of rodents), found exclusively in Kinmen where it dominated, *Rattus rattus mindanensis* (17% of rodents), found exclusively in Taitung where it also dominated, and *Rattus norvegicus* (21% of rodents), found at all sites [60]. For the aforementioned rodent and shrew species, the prevalence of chigger infestation ranged from 9% to 91%, averaging 35% across all species with chigger *O. tsutsugamushi* PCR positivity averaging 22% [60]. *O. tsutsugamushi* was detected by PCR in the spleens of 38% of rodents and 20% of shrews, and rodents (*n* = 690) had an overall seroprevalence of 69% [60]. Finally, Lin et al. [60] revealed a strong positive correlation (*r*^2^ = 0.83) between rodent seroprevalence and incidence of scrub typhus, in concordance with Kuo et al. [51].

#### 2.1.4. Genetics of Scrub Typhus

In 1995, Tamura et al. [69] proposed the reclassification of *Rickettsia tsutsugamushi* to a novel genus, *Orientia*, where *O. tsutsugamushi* remained the sole species until the discovery of a divergent species, *Orientia chuto*, isolated from an Australian tourist infected in the United Arab Emirates (UAE) in 2006 and described by Izzard et al. [70] in 2010, although no human cases have since been reported. Genotyping of *O. tsutsugamushi* is based on a genus-specific gene that encodes the 56-kDa type-specific antigen (*tsa56*), a major outer membrane protein comprising ~20% of the proteome, which demonstrates markedly high levels of diversity at four variable protein domains (VDI–VDIV) [71]. In a recent phylogenetic analysis of 206 complete or nearly complete *tsa56* sequences (≥1251 bases), Kim et al. [72] classified 17 existing genotypes into five genogroups (Karp, Gilliam, TA763, Kato, and Shimokoshi). Remarkably, 14 of 17 genotypes were identified in Taiwan, revealing the greatest genotypic diversity among the endemic countries, and identical sequences of Gilliam and Karp_A genotypes were identified in China, and in Thailand and Cambodia, respectively, while sub-identical (1 or 2 base difference) sequences of Boryong, Karp_A, and Kato_B genotypes were identified in Korea, Vietnam, and in Japan and Malaysia, respectively [72].

Lu et al. [29] isolated *O. tsutsugamushi* from the acute peripheral blood mononuclear cells (PBMCs) collected from 116 patients with scrub typhus throughout Taiwan from 2006 to 2007, mostly from Kinmen County (43 cases), Kaohsiung City (23 cases), and Nantou County (12 cases), although there were 1 to 6 cases from 11 additional counties. Complete *tsa56* sequences were obtained, representing 22 sequence types with >99.9% similarity, and phylogenetic analysis revealed that 9 sequence types belonged to Karp (69 isolates, 59%), 7 to Gilliam (21 isolates, 18%), 2 to TA763 (2 isolates), and 2 to Kato (4 isolates) genogroups (17 of 22 sequences included in Kim et al. [72]), also revealing two novel sequence types, TW-12 (1 isolate) and TW-22 (19 isolates) [29]. In a subsequent study, Lin et al. [57] classified TW-22 as Kato-related, and thus Lu et al. [29] identified 23 human isolates (20%) in the Kato genogroup. Lin et al. [57] obtained 68 complete *tsa56* sequences from ectoparasitic chiggers mostly collected between 2008 to 2009 in Hualien County (7), Taitung County (10), Kinmen (29), Matsu (3), Orchid (14), and the Pescadores Islands (5), and phylogenetic analysis revealed that 25 isolates belonged to Karp (37%), 18 to TA763 (26%), 13 to Kato (19%), and 12 to Gilliam (18%) genogroups (49 of 68 sequences included in Kim et al. [72]). Surprisingly, *tsa56* sequences in this field study [57] reflected the relative proportion of genogroups of human *tsa56* sequences in Lu et al. [29], with Karp being the most prevalent (37% versus 59%), then Kato (19% versus 20%), and Gilliam (18% in both studies), although TA763 had a large discrepancy (26% versus 2%).

Alternatively, PCR-restriction fragment length polymorphism (PCR-RFLP) analysis of *tsa56*, a PCR amplicon of the VDI region digested with *Hha*I or *Sfa*NI restriction enzymes [52], for example, produces differential restriction fragment sizes (an RFLP pattern) after gel electrophoresis that correlates to a 56-kDa genotype. Yang et al. [52] used this method to determine the *tsa56* genotype of 505 patients in eastern Taiwan, identifying Gilliam genogroup in a majority of patients (285, 56%), Karp in 155 (31%), Kato in 33 (7%), and TA763 genogroup in 30 patients (6%). However, a novel RFLP type ‘Taiwan-D’ identified in two patients did not have a corresponding *tsa56* sequence, although it was successfully isolated from one patient [52], so it could have been sequenced and later referenced. PCR-RFLP is a cost-effective and efficient technique for *tsa56* genotyping, although sequencing has become cheaper and more rapid.

### 2.2. Murine Typhus

#### 2.2.1. Epidemiology of Murine Typhus

In contrast with scrub typhus, studies describing murine (endemic) typhus in Taiwan are lacking. The Taiwan CDC NIDSS [32] reported 391 confirmed indigenous cases from 2004 to 2016, with an annual mean of 30 cases, occurring almost exclusively in western coastal districts/townships in the Kaohsiung-Pingtung region (213), Changhua County (52), Tainan City (41), and Taichung City (22). Chang et al. [53] only recently described the epidemiology and clinical manifestations of 81 confirmed cases of murine typhus that occurred in southern Taiwan between 1992 and 2009. Not unlike scrub typhus, males were more frequently infected with *R. typhi* than females (>2:1), although most cases occurred in those aged 50 to 59 (27%) with a mean age of 50 years, compared to 60 to 69 years for scrub typhus, while murine typhus cases similarly demonstrated a significant positive correlation with monthly average temperature (*r*^2^ = 0.747, *p* = 0.005) in most cases from May to August [53]. Likewise, Kuo et al. [73] recently analyzed murine typhus cases confirmed by the Taiwan CDC from 2000 to 2014, and revealed the highest IR in the 50 to 59-year age stratum and increased IR during May to August.

#### 2.2.2. Clinical Features of Murine Typhus

Symptoms of murine typhus include fever (96%), chills (61%), headache (52%), myalgia (28%), rash (28%), cough (26%), and relative bradycardia in 48 of 71 patients (68%) [53]. Chang et al. [50] later described 88 confirmed cases of murine typhus in southern Taiwan between 2002 and 2011 (ostensibly overlapping [53]), with a male-to-female ratio of >2:1 and with 22% elderly (≥65) versus 78% non-elderly (18 to 64 years). This study revealed significant differences (*p* <0.05) in clinical presentations in non-elderly versus elderly patients, with higher prevalence of fever (100% versus 84%), chills (67% versus 35%), headache (61% versus 23%), and cough (32% versus 6%), whereas the elderly had a higher prevalence of dyspnea (24% versus 3%) [50]. Chang et al. [53] reported ARF in 5 of 81 patients (6%), meningitis in 3 patients (4%), and ARDS in 2 patients—resulting in 1 fatality, with a case fatality rate of 1%. Nonetheless, murine typhus is usually self-limiting and uncomplicated [74], and it is estimated that up to 80% of cases are undiagnosed [75].

#### 2.2.3. Ecology of Murine Typhus

*R. typhi* is maintained in the classic rat–flea cycle by commensal rodents, primarily *Rattus* species, with the Oriental rat flea (*Xenopsylla cheopis*) as the principal vector [76]. *X. cheopis* has also been demonstrated to maintain *R. typhi* through vertical transmission (transstadially and transovarially) [76]. Horizontal transmission to vertebrate hosts may occur when infected flea feces or tissues contaminate broken skin (at the site of the bite), conjunctiva, or the respiratory tract [76]. Due to the ubiquity of *X. cheopis* and *Rattus* species [49], murine typhus is distributed throughout the globe as one of the most prevalent type of rickettsiosis [74]. Murine typhus most commonly occurs in warm coastal areas where rodents and their fleas flourish [77], explaining the distribution of murine typhus in Taiwan (i.e., in western coastal districts/townships). In fact, in a geographically weighted regression model, Kuo et al. [73] found that risk for murine typhus increases with increasing proximity to operating seaports, particularly in districts near Kaohsiung and Taichung international seaports. Chien et al. [67] conducted a field survey of small mammals and their ectoparasites at international ports around Taiwan and its offshore islands from 2004 to 2011. Of the 350 animals that were seropositive for *R. typhi*, 316 of them (90%) were of *R. norvegicus*, 221 of which were captured at Kaohsiung Harbor, 19 at the nearby Kaohsiung International Airport, 50 at the Taichung Harbor, and 16 at the Mailiao Harbor in Yunlin, representing *R. norvegicus* seropositivity rates of 32%, 22%, 24%, and 7%, respectively, while all other international ports revealed overall *R. typhi* seropositivity rates between 0 and < 2% [67]. Further, *X. cheopis* represented 79%, 94%, 74%, and 52% of all ectoparasites collected from animals at Kaohsiung Harbor, Kaohsiung International Airport, Taichung Harbor, and Mailiao Harbor, respectively, although they were not assayed for *R. typhi* [67]. Kuo et al. [61] similarly trapped small mammals and collected their fleas in eastern Taiwan during 2007 and 2008, and the overall seropositivity rate for *R. typhi* was 0.1%, detected in just one animal (*Apodemus agrarius*), while only two flea species were identified (*Stivalius aporus* and *Acropsylla girshami* Traub), with no *R. typhi* DNA detected. These results are not surprising, as no cases of murine typhus have been confirmed in the townships in which this study was conducted, according to the Taiwan CDC NIDSS [32].

### 2.3. Co-Endemic Diseases and Co-Infection

Indeed, both murine typhus and scrub typhus (and other VBR) consistently present with fever, while other symptoms are non-specific and inconsistent, making early differential diagnosis difficult. Further, other co-endemic diseases such as acute Q fever and leptospirosis, bacterial zoonoses caused by *Coxiella burnetii* (a rickettsia-like organism [74]) and *Leptospira* spirochetes [78], respectively, also cause acute undifferentiated fever and complicate differential diagnosis. Depending on the year and region, these co-endemic diseases can occur more frequently than VBR. Acute Q fever had an annual mean of 42 confirmed indigenous cases from 2011 to 2016, compared to 111 from 2004 to 2010, while there was a mean of 82 cases of leptospirosis confirmed annually from 2004 to 2016 [32]. Although rare, co-infection of VBR with these bacterial zoonoses has been observed in Taiwan. Lai et al. [79] analyzed Taiwan CDC data from 2007 to 2014 and identified co-infection of acute Q fever with scrub typhus (7 cases), murine typhus (4 cases), and leptospirosis (4 cases), also identifying co-infection of scrub typhus with leptospirosis (11 cases). Strikingly, of 11,109 cases suspected of acute Q fever or co-infection with scrub typhus, murine typhus, or leptospirosis (or mixed infection) reported to the Taiwan CDC, only 13% of cases were confirmed (1420) [79].

## 3. Emerging Vector-Borne Rickettsioses

### 3.1. Spotted Fever Group

To date, over 20 novel *Rickettsia* species belonging to the tick-borne SFG have been identified throughout the world, known to cause a myriad of diseases, most notably Rocky Mountain spotted fever caused by *Rickettsia rickettsii* [4,49,80]. In Taiwan, however, no human SFG rickettsioses (SFGR) have been confirmed, although Lai et al. [17] recently conducted a retrospective serological study and identified 2 patients acutely infected with undetermined SFGR and 10 patients with past infection. Specifically, paired sera from 413 patients with acute undifferentiated fever (255 cases of unknown etiology) seen from 2004 to 2009 in a hospital in Kaohsiung was screened using IFA against *R. rickettsii* (IgG and IgM), and, if positive, subjected to a micro-immunofluorescence (MIF) tests against *R. rickettsii*, *Rickettsia conorii*, *Rickettsia japonica*, *R. felis*, and *R. typhi* (IgG and IgM) [17]. Both cases determined to be acutely infected with SFGR had unknown etiology after being screened at Taiwan CDC, representing 2 of 255 cases of unknown etiology for acute undifferentiated fever included in this study [17]. Despite the limited number of cases with unknown etiology screened for SFGR in this study, Lai et al. [79] identified that a staggering 87% of cases screened for clinically suspected acute Q fever, scrub typhus, murine typhus, and leptospirosis reported to the CDC from 2007 to 2014, were negative, representing 9,689 cases of unknown etiology. Based on the findings of Lai et al. [17], it is not unreasonable to postulate that SFGR caused some of these acute undifferentiated febrile illnesses.

#### Ecology of SFG Rickettsiae

Similar to *O. tsutsugamushi*, a rich diversity of SFG rickettsiae has been observed throughout Taiwan in nature, with several studies having examined small mammals and their ticks (Table 1). Tsui et al. [63] were the first to isolate and molecularly identify SFG rickettsiae in Taiwan, identifying *Rickettsia* sp. TwKM01 (*Rickettsia rhipicephali* cluster) in *Rhipicephalus (Rh.) haemaphysalodies* ticks collected from rodents in Kinmen and Hualien, and *Rickettsia* sp. TwKM02 (*Rickettsia australis* cluster) in *L. deliense* collected from rodents in Kinmen and Matsu. While *R. rhipicephali* is an unlikely human pathogen [81], *R. australis* (TRG) is the etiologic agent of Queensland tick typhus [82], which occurs primarily in northeastern Australia [49]. Tsai et al. [64] then isolated a novel SFG rickettsiae, *Rickettsia* sp. IG-1, from *Ixodes granulatus* ticks collected from Orchid Island, validated by criteria put forth by Fournier et al. [83]. Perhaps unsurprisingly, Hsu et al. [54] identified *Rickettsia* sp. RR01 (*R. rhipicephali* cluster) in 7% of adult *Rhipicephalus sanguineus* and *Rh. haemaphysaloides* ticks collected from stray dogs in a Taichung animal shelter in 2008. Kuo et al. [65] revealed markedly higher *R. conorii* seroprevalence (92%) compared to the seroprevalence of *O. tsutsugamushi* (70%) in rodents collected in eastern Taiwan during 2007 and 2008, also detecting *Rickettsia* sp. TwKM01, *R. conorii*, and *R. japonica* DNA in *S. aporus* fleas [61], representing the first reports of *R. conorii* and *R. japonica* in Taiwan. *R. conorii* subspecies *conorii*, *caspia*, and *israelensis* are known to cause Mediterranean spotted fever, Astrakhan fever, and Israeli spotted fever, respectively, which are clinically similar, while *R. japonica* causes Japanese spotted fever [74]. In another arm of the study described above, Kuo et al. [62] detected *Rickettsia* sp. TwKM01, *Rickettsia* sp. IG-1, *R. conorii*, and *R. japonica* in ticks collected from small mammals, also detecting *R. rickettsii* in *I. granulatus* from Kinmen and Matsu and *Rh. haemaphysaloides* from Kinmen, as well as *R. australis* in *I. granulatus* from Kinmen. Further, small mammals had *R. conorii* or *R. rickettsii* seroprevalence of 86%, with 64% to 96% seropositivity among study sites, and SFG rickettsiae DNA was detected in 41% of all animals, including the previously mentioned species (except *R. australis*), *Rickettsia* sp. TwKM02 in Yilan, and *Rickettsia raoultii* in Yilan, Taitung, and Taichung [68]. Of note, *R. raoultii* causes tick-borne lymphadenopathy and *Dermacentor*-borne necrosis and lymphadenopathy [77]. Kuo et al. [84] recently identified *Rickettsia helvetica* in eight individual *Ixodes columnae* larva collected from four local bird species in eastern Taiwan and one migratory bird species captured from 1995 to 2016, also identifying *Rickettsia monacensis* in one *Ixodes nipponesis* collected from one local bird species. *R. helvetica* causes an atypical rickettsiosis similar to *R. raoultii* [77], and *R. monacensis* has been documented to cause an uncharacterized rickettsiosis in North Africa and Korea [49].

### 3.2. Rickettsia felis

*R. felis* is an emerging flea-borne rickettsiosis known to cause flea-borne spotted fever, with cases documented throughout the world; however, its transmission biology remains poorly understood [85,86]. Since its discovery in a laboratory colony of *C. felis* in 1990 [87], *R. felis* has been detected in a myriad of invertebrate and vertebrate hosts, including over 40 arthropod species (e.g., fleas, ticks, mites, and mosquitoes) [88] and a variety of animals (e.g., dogs, cats, horses, rodents, opossums, and hedgehogs) [89,90].

#### 3.2.1. Clinical Features of *R. felis* Rickettsiosis

The first indigenous human case of *R. felis* rickettsiosis in Taiwan was identified in 2005 in a 27-year-old female woman residing in Kaohsiung [16], representing one of very few molecularly-confirmed cases of human *R. felis* rickettsiosis worldwide [77]. This patient presented with an acute undifferentiated febrile illness with chills, headache, fatigue, and acute polyneuropathy, and was treated with doxycycline for suspected zoonosis, which resolved all symptoms, and *R. felis* rickettsiosis was later confirmed by the Taiwan CDC with quantitative PCR (qPCR) and IFA [77]. Lai et al. [17] identified three acute cases of *R. felis* rickettsiosis by MIF among two patients co-infected with acute Q fever and scrub typhus, respectively, and one patient with previously unknown etiology. These patients similarly presented with fever, chills, headache (2), and all three patients had relative bradycardia, while other symptoms may be attributed to comorbidities. This study [17] identified three additional patients with past history of *R. felis* infection determined by MIF.

#### 3.2.2. Ecology of *R. felis*

Tsui et al. [63] first isolated *R. felis* from *L. deliense*, *I. granulatus*, and Mesostigmata mite pools collected from small mammals in Kinmen, Matsu, and Hualien, respectively. Tsai et al. [55] then isolated *R. felis* from *C. felis* (i.e., the principal vector and reservoir) collected from domestic and stray dogs and cats in northern Taiwan, observing higher rates of infection in *C. felis* collected from stray animals than domestic ones. However, this does not represent the first known incidence of *R. felis*-infected *C. felis* in Taiwan, as Tsai et al. [56] found 18% *R. felis* PCR positivity in 40 75% ethanol-preserved samples of *C. felis* collected from Taipei in 1991. Maina et al. [91] also detected *R. felis* DNA in *C. felis* collected from Orange County, California from 1969 to 1988. Together, these findings suggest that *R. felis* was circulating throughout the world prior to its discovery in 1990.

More studies in Taiwan focused on investigating the prevalence of *R. felis* in *C. felis* parasitizing cats and dogs, in order to elucidate the potential risk for *R. felis* to infect humans, particularly in an urban setting. Hsu et al. [54] examined *C. felis* collected from stray dogs and cats in Taipei in 2006, identifying *R. felis* in 70 of 158 (44%) *C. felis* pools with a minimum infective rate (MIR) of 16%. Tsai et al. [56] identified *R. felis* in 20% and 23% of individual *C. felis* samples collected from stray cats and dogs, respectively, in Taipei during 2006 and 2007. In an unpublished study, we found *R. felis* seropositivity of 30% in cats and 70% in dogs in Taipei City during 2011 and 2012. Kuo et al. [61] detected *R. felis* in *S*. *aporus* and *A. episema* fleas collected from rodents in Hualien during 2007 and 2008. Although this study did not collect *C. felis* in eastern Taiwan, we collected 93 *C. felis* samples from domestic dogs at an animal hospital in Hualien during 2015, also finding *R. felis* PCR positivity of 14% (unpublished). In the study describing scrub typhus [51] and SFG rickettsiae [62,68] throughout Taiwan, Kuo et al. detected *R. felis* DNA in *I. granulatus* collected in Kinmen, also detecting *R. felis* DNA in rodents collected from Taitung, Taoyuan, Matsu, Kinmen, and Penghu study sites, indicating widespread distribution.

### 3.3. Anaplasmataceae

The Anaplasmataceae are emerging tick-borne disease agents, including *Ehrlichia chaffeensis* and *Ehrlichia ewingii*, which cause human monocytic and granulocytic ehrlichiosis, respectively, and *Anaplasma phagocytophilum*, the etiologic agent of human granulocytic anaplasmosis [74]. While no cases of human ehrlichiosis or anaplasmosis have been identified in Taiwan, *A. phagocytophilum* and *E. chaffeensis* have been detected in domestic animals (i.e., dogs and horses) and in small mammals.

Liu et al. [92] revealed markedly low *A. phagocytophilum* PCR positivity of 2% in 110 dogs surveyed in Pingtung during 1999 and 2000. In sera collected from 62 domestic dogs in northern Taiwan during 2008, nearly a decade later, *A. phagocytophilum* seropositivity was similarly 2% [93]. Then, in a 2009 survey of horses throughout Taiwan’s western coast, from Taipei to Pingtung, overall *A. phagocytophilum* seroprevalence was again found to be 2% [94]. However, it is important to note that the commercial kit used in these serological studies demonstrates cross-reactivity between *A. phagocytophilum* and *Anaplasma platys* [95], potentially overestimating *A. phagocytophilum*. However, Masuzama et al. [96] detected *A. phagocytophilum*-specific DNA in the spleens of 6 rodents (16%) collected from Kinmen in 1999, 1 rodent (2%) from Taichung in 1999, and 5 rodents (9%) from Taichung in 2009.

Weng et al. investigated ticks parasitizing rodents throughout Kinmen in 2009 for *E. chaffeensis*, revealing a MIR of 2% in two pools of *Rh. haemaphysaloides* and *I. granulatus* nymphs collected from *R. losea exiguous* [97]. Then, in 2012, they detected *E. chaffeensis* DNA in the livers or spleens of 15% of rodents captured throughout Kinmen [98]. Tsai et al. [66] collected 1,648 small mammals throughout the main island of Taiwan from 2004 to 2008, detecting *E. chaffeensis* antibodies in 54 (3%), mostly in northern Taiwan, while no seropositive animals were detected in eastern Taiwan.

## 4. Research Gaps and Future Directions

### 4.1. Scrub Typhus

The recent emergence of *O. chuto* in the UAE [70] and the broader emergence of scrub typhus (e.g., in Chile and Africa [99]) outside of its area of endemicity, the Asian-Pacific ‘tsutsugamushi triangle,’ taken together with the re-emergence and diversification [72] of scrub typhus in Taiwan, underscores the importance of Taiwan in the international study of scrub typhus.

#### 4.1.1. National Scrub Typhus Genotyping Surveillance

National scrub typhus genotyping surveillance (NSTGS) would greatly improve our collective understanding of scrub typhus, with global implications. Important research questions could be answered, such as:

- How do scrub typhus genotypes vary by space and time (i.e., by region and season)?
- Do clinical manifestations vary by scrub typhus genotype (e.g., signs and symptoms)?

NSTGS could be implemented in any scrub typhus-endemic country with a national communicable disease surveillance system (e.g., the NNDSS in Taiwan). For instance, the Taiwan CDC could request acute phase whole blood from patients with suspected scrub typhus and perform nested PCR to amplify the VDI region of *tsa56*, then genotyping could be accomplished by either direct sequencing or subsequent RFLP analysis, as performed by Yang et al. [52]. Then, genotype and/or genogroup could be reported with each confirmed case of scrub typhus through an open database such as the NIDSS.

#### 4.1.2. Antibiotic-Resistant Scrub Typhus

The recent emergence of antibiotic-resistant scrub typhus (ABR-ST) is alarming, yet seriously neglected. Watt et al. [100] first reported chloramphenicol- and doxycycline-resistant scrub typhus among patients in Chiangrai, northern Thailand in 1996. Tanskul et al. [101] then conducted an ecological and epidemiological investigation in the villages of patients infected by the resistant strains. In search of an alternative therapy, Watt et al. [102] compared azithromycin and doxycycline efficacy against three of the resistant strains in mice, revealing no significant difference between the two treatment groups for mice infected with the resistant strains; however, they observed a marked response to rifampicin [103]. Therefore, they conducted a single-blind randomized trial to compare the efficacy of treatment with 200 mg doxycycline, 600 mg rifampicin, or 900 mg rifampicin [103]. They revealed significantly shorter (*p* = 0.01) fever clearance (defervescence) in those treated with 900 mg and 600 mg rifampicin, with 22.5 and 27.5 h medians, respectively, compared to a 52 h median in those treated with 200 mg doxycycline, also observing significantly higher proportions (*p* = 0.02) of those achieving defervescence within 48 h in the rifampicin regimens [103]. Although rifampicin is efficacious against chloramphenicol- and doxycycline-resistant strains of *O. tsutsugamushi*, it is likewise possible for rifampicin resistance to develop, as it is conferred by a single point mutation in the RNA polymerase β-subunit encoding gene (*rpoB*) [104]. Thus, alternative therapies must be evaluated to treat resistant scrub typhus.

Alarmingly, delayed defervescence (i.e., fever lasting >3 days) after doxycycline treatment was identified in seven patients with scrub typhus in a hospital-based retrospective study [105] in southern Taiwan published in 2009; however, it is unknown whether these cases were caused by doxycycline resistance. Since evaluating ABR-ST requires high containment (i.e., biosafety level 3) facilities, using PCR techniques (e.g., TaqMan-based qPCR) to detect genes that confer resistance (e.g., rpoB) in acute PBMCs is a practical alternative for identifying ABR-ST. Liao et al. [106] recently reported the complete genomes of *O. tsutsugamushi* strains AFSC4 and AFSC7 isolated by Watt et al. with doxycycline, chloramphenicol, and azithromycin resistance, and *O. tsutsugamushi* Karp, which is susceptible to these antibiotics. Genome comparison revealed two hypothetical proteins present in the genomes of AFSC4 and AFSC7 that were absent in the Karp genome, potentially representing genes that confer antibiotic resistance. Identifying such genes is a critical first step towards developing ABR-ST genotyping, which may one day guide precision medicine.

Interestingly, in 1990, Hasegawa et al. [107] postulated that the misuse of antibiotics may have given rise to divergent scrub typhus genotypes. Perhaps an *in vivo* study could investigate whether the repeated use of tetracycline-class antibiotics drives mutation or recombination at the *tsa56* locus, providing insight towards understanding the acquisition of antibiotic resistance in scrub typhus.

#### 4.1.3. Investigation of Migratory Birds

The potential for migratory birds in the spread of *O. tsutsugamushi* genotypes throughout the Asian-Pacific region has been speculated for decades [108], and, more recently, postulated as a mechanism for the spread of scrub typhus to other parts of the world [99]. Kim et al. [72] clearly demonstrated the convergence of the East Asian-Australasian migratory bird flyway in Taiwan. Notably, Taiwan’s outermost islands neighboring the southeastern coast of China (i.e., Kinmen and Matsu Islands) are in the direct path of this flyway. Also, as demonstrated here, the Kinmen and Matsu Islands harbor a rich diversity of *O. tsutsugamushi* genotypes and chigger species, respectively. Indeed, additional studies are needed to validate whether migratory birds facilitate the spread of *O. tsutsugamushi* genotypes regionally and intercontinentally, and should include molecular characterization of *O. tsutsugamushi* infecting the birds, analysis of ectoparasitic chiggers, and follow-up ecological surveillance in their destinations, if feasible.

### 4.2. Murine Typhus

It is unclear whether an urban cycle [109] exists for murine typhus in Taiwan, thus necessitating future investigation. In particular, the potential role of domestic animals (i.e., dogs and cats) should be examined, as *R. typhi* DNA has been detected in dogs in Mexico [110], in cats in Spain [111], and in *C. felis* collected from dogs in the U.S. [112]. Also of note, 17 of 81 patients (21%) with murine typhus in southern Taiwan from 1992 to 2009 described by Chang et al. [53] reported recent close contact with cats or dogs. Perhaps studies in domestic animals could help explain the sporadic cases observed in non-coastal districts/townships along Taiwan’s west coast.

### 4.3. Emerging Vector-Borne Rickettsioses

The burden of emerging VBR in Taiwan is unknown. Typically, once a physician diagnoses a patient with a rickettsial-like illness, the patient is treated with doxycycline or another tetracycline-class antibiotic, and the illness resolves. Although biological specimens are delivered to the Taiwan CDC for screening, the etiologic agent is not identified in a vast majority of cases [79]. Meanwhile, veterinarians often rely on rapid commercial tests that detect a panel of cross-reactive species, so again the exact pathogen is not identified. Importantly, many emerging VBR demonstrate cross-species virulence (e.g., *A. phagocytophilum*), and thus infection in either a human or domestic animal represents a ‘shared risk’ [113]. Accordingly, in both settings, an attempt should be made to identify the species. In the case that an emerging VBR is detected, local physicians and veterinarians should communicate the sentinel event to one another, in addition to alerting public health authorities, and enlist environmental health scientists (e.g., entomologists, zoologists, and epidemiologists) to initiate an interdisciplinary investigation.

While it is not feasible to adopt all VBR as notifiable diseases, as hospitals may not have access to the appropriate diagnostic tools needed for their differential diagnosis, physicians suspecting VBR should include these as suspected diseases when delivering biological specimens to the Taiwan CDC, as they are capable of screening cases for non-notifiable VBR (i.e., SFGR and *R. felis*). We respect that some research institutions are capable of performing these analyses on their own. Further, the Taiwan CDC should attempt isolation if an emerging VBR is detected, as immunodominant proteins from local strains (e.g., *Rickettsia* sp. IG-1) can be used to develop rapid diagnostics. It is equally important to isolate novel strains of *O. tsutsugamushi* (e.g., *O. tsutsugamushi* TW-12), as antigens from such strains can be used to improve rapid tests that physicians can use to promptly diagnose and treat patients. Veterinarians can collaborate with research institutions to identify the pathogen when a zoonotic agent is suspected.

Existing epizoological studies on the Anaplasmataceae in Taiwan could provide more insight by identifying pathogens based on multiple gene targets. These studies tend to sequence the 16S rRNA gene (*rrs*) exclusively [92,96,97,98,114], but phylogenetic analysis is strained as *rrs* is highly conserved for some species [115], so additional gene targets such as *groEL* [95] should be considered. Similarly, studies investigating emerging rickettsiae tend to sequence a single gene target (e.g., *gltA* [54] or *ompB* [114]); however, it is difficult to parse species based on one sequence as increasingly more sequences are made available. Thus, researchers should consider sequencing multiple gene targets to accurately identify emerging VBR.

## 5. Conclusions

Scrub typhus and murine typhus were studied in Taiwan amidst their discovery in the early twentieth century by Japanese scholars; however, murine typhus has remained largely neglected in Taiwan since WWII, even to this day. While scrub typhus was later studied by the U.S. Navy during the mid-late twentieth century, it received little attention until the past decade, after its re-emergence. Despite the increasing numbers of studies on scrub typhus in Taiwan and elsewhere, many critical questions outlined in this review remained unanswered, which are becoming increasingly urgent in light of the global spread of scrub typhus.

Alarmingly, mounting evidence demonstrates the emergence of VBR such as SFGR and *R. felis* rickettsiosis in Taiwan. Through this review, it is clear that emerging VBR in Taiwan remain underappreciated. Thus, increasing the awareness of emerging VBR among physicians and veterinarians is a critical first step towards establishing integrated surveillance to detect these pathogens. Moreover, interdisciplinary collaboration is essential to understanding and controlling the spread of these potentially life-threatening diseases.

## Figures and Tables

**Table 1:** Clinical and ecological evidence of re-emerging and emerging human VBR in Taiwan.

		Scrub Typhus Group	Typhus Group	Spotted Fever Group	Transitional Group
		*O. tsutsugamushi*	*R. typhi*	*Rickettsia* spp.	*R. felis*
**NNDSS, Taiwan CDC**		notifiable		non-notifiable	
**humans**		Lu, 2010 ^1,2,3^ [29] Yang, 2012 ^1,2,3^ [52]	Chang, 2012 ^1^ [53]	Lai, 2014 ^1^ [17]	Tsai, 2008 ^1,2^ [16] Lai, 2014 ^1^ [17]
**arthropod ectoparasites**	domestic animals			Hsu, 2011 ^2^ [54]	Tsai, 2009 ^2,3^ [55] Hsu, 2011 ^2^ [54] Tsai, 2011 ^2^ [56]
	Small mammals	Lin, 2011 ^2^ [57] Kuo, 2011 ^2^ [58] Kuo, 2012 ^2^ [59] Lin, 2014 ^2^ [60] Kuo, 2015 ^2^ [51]	Kuo, 2012 ^2^ [61] Kuo, 2015 ^2^ [62]	Tsui, 2007 ^1,2,3^ [63] Tsai, 2008 ^1,2,3,^* [64] Kuo, 2012 ^2^ [59] Kuo, 2012 ^2^ [61] Kuo, 2015 ^2^ [62]	Tsui, 2007 ^1,2,3^ [63] Kuo, 2012 ^2^ [61] Kuo, 2015 ^2^ [62]
**small mammals**		Kuo, 2011 ^1^ [65] Lin, 2014 ^1,2^ [60] Kuo, 2015 ^1^ [51] Tsai, 2016 ^1^ [66]	Chien, 2012 ^1^ [67] Kuo, 2012 ^1^ [61] Kuo, 2015 ^1,2^ [68]	Kuo, 2011 ^1^ [65] Kuo, 2012 ^1^ [61] Kuo, 2015 ^1,2^ [68]	Kuo, 2015 ^1,2^ [68]

^1^ indirect immunodetection (e.g., IFA); ^2^ direct molecular detection (e.g., PCR); ^3^ isolation (shell vial centrifugation); * novel species.
